# Genetic variants and carotid atherosclerosis progression in familial hypercholesterolemia: a comprehensive review

**DOI:** 10.3389/fcvm.2025.1526093

**Published:** 2025-05-29

**Authors:** Makhabbat Bekbossynova, Tatyana Ivanova-Razumova, Yerkin Azatov, Aliya Sailybayeva, Sadyk Khamitov, Gulnur Daniyarova, Kamila Akzholova

**Affiliations:** ^1^ “University Medical Center” Corporate Fund, Astana, Kazakhstan; ^2^ Pediatric Clinical Academic Department, “University Medical Center” Corporate Fund, Astana, Kazakhstan; ^3^ Cardiology Department № 2, “University Medical Center” Corporate Fund, Astana, Kazakhstan; ^4^ Research Department, “University Medical Center” Corporate Fund, Astana, Kazakhstan; ^5^ Nazarbayev University School of Medicine, Astana, Kazakhstan

**Keywords:** familial hypercholesterolemia, carotid atherosclerosis, APOB, LDLR, cIMT

## Abstract

**Introduction:**

Familial Hypercholesterolemia is a hereditary metabolic disorder characterized by elevated low-density lipoprotein cholesterol, as high as 1 in 250 individuals, leading to cardiovascular diseases like atherosclerosis. It's caused by autosomal dominant mutations in genes LDL receptor, Apolipoprotein B-100, and proprotein convertase subtilisin/kexin type 9 (PCSK9), FH leads to lifelong elevation of LDL-C. Carotid atherosclerosis, a sign of systemic atherosclerosis, can be studied as a clinical feature of FH, providing insights into its risk assessment, early diagnosis, and intervention.

**Objective:**

To determine contribution of specific genetic variants to carotid atherosclerosis, thereby improving our understanding of the genetic basis of cardiovascular risk in FH.

**Methods:**

A search was performed through PubMed, Google Scholar, Medline and Scopus databases using the preselected terms. Studies were selected and reviewed based on inclusion and exclusion criteria by two authors independently, with third-party adjudication.

**Results:**

Total of 9 trials were included: 4 cross-sectional studies, 4 retrospective cohorts and 1 prospective cohort studies. Total sample size of all reviewed studies was 3,033 in different settings. Studies revealed higher cIMT levels in FH patients and showed significant association of LDLR mutations with severe atherosclerosis. APOB and PCSK9 mutations in this study had limited effect on cIMT levels and prevalence of carotid plaques.

**Conclusion:**

This review highlights the essential role of LDLR mutations in progression of carotid artery atherosclerosis among patients with FH. Incorporating information on FH mutations into risk assessment for atherosclerosis patients can help predict disease progression and cardiovascular outcomes more effectively.

## Introduction

Familial hypercholesterolemia (FH) is one of the underdiagnosed conditions with autosomal dominant inheritance, globally affecting 1 in every 250 people worldwide (1, 2). Clinically, FH is manifested with high levels of low-density lipoprotein cholesterol (LDL-C) which predisposes patients to arcus corneae, formations of xanthomas, to premature atherosclerosis and cardiovascular diseases (CVD) (2, 3). The genetic origin of FH is attributed to the mutations in lipid metabolism related genes, such as low-density lipoprotein receptor gene (LDLR), apolipoprotein B (APOB) and proprotein convertase subtilisin/kexin type 9 (PCSK9) (1, 4). As a result of impaired function of LDLRs fewer molecules of LDLC are removed from the blood by the liver, leading to hypercholesterolemia (5). Consequently, severe, and life-long elevation of LDL-C in patients carrying respective mutations increases the risk of atherosclerotic cardiovascular disease (ASCVD) (5).

For more than 50 years, the prevalence of FH in the general population was considered to be about 1:500, however, recent studies estimated it to be as high as 1:313 in among 11 million subjects in 2020 (3, 6). Similarly, Hu and colleagues reported the FH prevalence of 1:311 in their 2020 study (7). These indicators were further updated by Fularski et al. with frequency of FH to be 1:250 (2). These estimates are particularly alarming given that people with FH have a 13-fold increased risk of CHD and that, in the absence of therapy or intervention, only 20% of FH patients will live to be 70 years old (8, 9). Studies show that both ASCVD events and CVD-related mortality can be decreased with early diagnosis and intervention in FH patients (10, 11).

Studies also highlight the presence of autosomal recessive type of FH (HoFH) which is mainly driven by homozygous mutations in LDL-R protein (1). In comparison with FH, the frequency of HoFH is lower as 1:250,000 and 1:360,000 and has phenotypic manifestation in early childhood (1, 2). Despite the higher prevalence of FH than HoFH, FH is characterized to be silent disease and generally underdiagnosed and thus largely being untreated (1, 2).

Atherosclerosis, known as a leading mortality cause in developed countries, is characterized by thickening of arterial walls due to the accumulation and build-up of fat and cholesterol plaques (12). Accumulation of plaques in carotid arteries, subsequently causing carotid artery atherosclerosis, is a sign of systemic atherosclerosis and one of the main predictors of CVDs such as myocardial infarction and ischemic stroke (13). Carotid intima media thickness (C-IMT) is frequently used as a measure of carotid artery atherosclerosis and it can be detected and measured using carotid ultrasonography (14). Since the individuals with FH are under the lifetime exposure to elevated LDL-C, the progression of carotid artery atherosclerosis within this category of patients are in high concern.

In contrast to the well-established link of FH and cardiovascular risk, the effects of individual genetic abnormalities within the FH population on atherosclerosis progression still remains underexplored. Taking into account varying effects of each LDLR, APOB and PCSK9 mutations on LDL-C levels, understanding the course of carotid atherosclerosis in patients with FH with unique mutations may be informative for assessing risk and starting early interventions and eventually lessen the cardiovascular load in patients with FH.

The purpose of this systematic review is to summarize existing evidence on the effects of LDLR, ApoB, and PCSK9 mutations on carotid artery atherosclerosis progression in FH population. By comparing the atherosclerotic burden in FH patients with these mutations to healthy controls, this study aims to determine the extent to which specific genetic variants contribute to carotid atherosclerosis, thereby improving our understanding of the genetic basis of cardiovascular risk in FH.

## Methods

### Study design

The current systematic review was conducted in accordance with Preferred Reporting Items for Systematic Reviews and Meta-Analyses (PRISMA) guidelines. The primary objective of this study was to examine the influence of LDLR, ApoB, and PCSK9 gene variants on the progression of carotid artery atherosclerosis in people with FH to healthy controls.

### Search strategy

The searching of relevant literature was undertaken across electronic databases, particularly PubMed, Google Scholar, Medline, and Scopus. The combination of MeSH terms and free text keywords associated with FH, LDLR, APOB, PCSK9 mutations and related to carotid artery atherosclerosis were applied in the search strategy. Additional reference searches of bibliography of relevant articles were conducted to identify relevant studies. The last search was made on 10 August 2024. The following search terms were used to conduct systematic search:

- (“familial hypercholesterolemia” OR “FH” OR “high LDL-C” OR “hypercholesterolemia”) AND (“LDLR mutations” OR “LDLR gene” OR “ApoB mutations” OR “ApoB gene” OR “PCSK9 mutations” OR “PCSK9 gene”) AND (“carotid artery atherosclerosis” OR “carotid intima-media thickness” OR “CIMT” OR “carotid plaque”).
- (“familial Hypercholesterolemia” OR “FH”) AND (“carotid artery Atherosclerosis” OR “carotid Atherosclerosis”) AND (“LDL-C” OR “APOB” OR “PCSK9”)

### Inclusion and exclusion criteria

Titles and abstracts of identified studies were screened for relevance, and full-text articles were reviewed for eligibility by two authors. The inclusion criteria for this review encompass randomized controlled trials (RCTs), observational studies, cohort studies, case-control studies, and cross-sectional studies that include:

- Studies with 18–65 years patients who have genetically verified FH diagnosis.
- Studies exploring FH genetic mutations (LDLR, APOB, or PCSK9)
- Studies on carotid artery atherosclerosis (measured by CIMT, presence of plaques)
- Studies with control groups without FH and normal LDL-C levels.

While following studies for excluded from final stage analysis:

- Studies involving patients with hypercholesterolemia not specifically diagnosed as FH
- Studies without clear definitions or results for the group of FH patients
- Studies involving patients outside of 18–65 age range (pediatric patients)
- Animal studies, *in vitro* studies, and studies that do not use genetic testing to confirm FH diagnosis are excluded.
- Reviews and case study papers, as well as editorials and conference abstracts
- Studies in languages other than English

### Data extraction

Data was extracted independently by two reviewers, with discrepancies resolved through discussion and third-party adjudication. The data were extracted in the following format below:

- Study characteristics (author, year, study design, sample size)
- Participant characteristics (age, gender, LDL-C levels, genetic mutation)
- Outcome measures (CIMT, plaque presence, progression of atherosclerosis).

## Results

### Study selection

**Figure 1:**
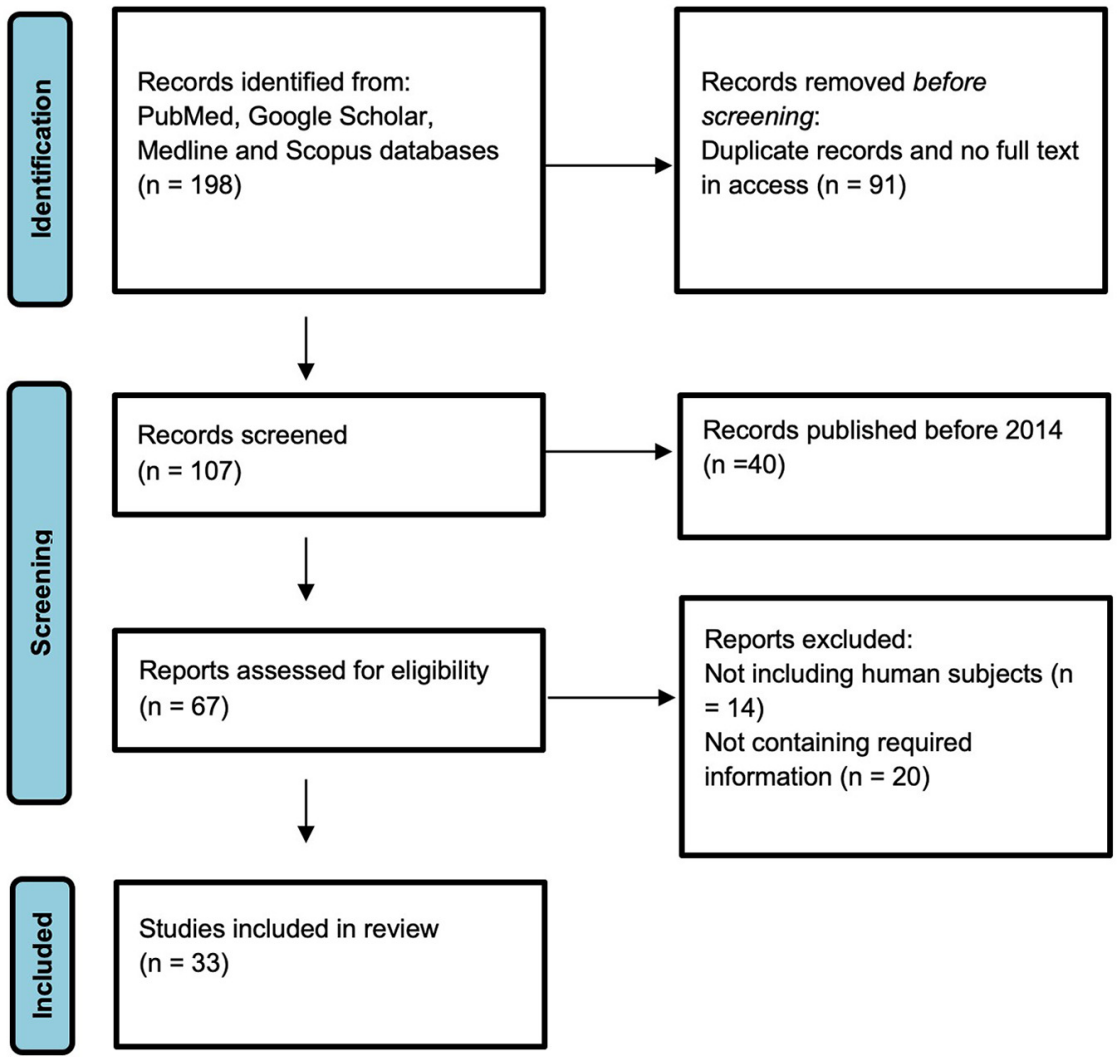
Study selection process.

The results of the study selection process are summarized by Figure 1. At the initial stage of study selection 198 studies were identified. After the removal of duplicate studies, titles and abstracts of 107 studies were screened for relevance. After title and abstract screening, 67 studies were selected for full-text review. Finally, 9 papers that met inclusion criteria were included into the final systematic review with overall 33 scientific articles used in the study.

### Study characteristics

Out of the 9 studies that met inclusion criteria and were included in this systematic review, 4 were cross-sectional studies, 4 were retrospective cohort studies and one study had prospective cohort study design. Total sample size of all reviewed studies was 3,033 individuals with 2,819 FH patients and 214 healthy controls. These studies had different settings which included Spain, Italy, Netherlands, Romania, Japan, Russia, and China. Variations in statin and PCSK9 inhibitor use among the study populations may have influenced the observed cIMT differences. Some studies adjusted for lipid-lowering therapy, while others did not, making direct comparisons challenging (15). Future studies should stratify findings by treatment exposure to isolate genetic effects. The overall eligible age range for most of the studies was within the 18–75 age range with the average age of 40 ± 15 years. Out of 9 studies, 8 studies investigated LDLR mutations, 7 studies focused on APOB mutations, while investigation of PCSK9 mutations were included in 6 studies (Table 1).

**Table 1:** Summary of reviewed studies.

Authors	Setting	Study design	Mutation type	Sample size	Primary outcomes	Key findings
Junyent et al. 2005 (23)	Spain	Cross-sectional study	LDLR	436	Carotid IMT	Higher IMT in FH patients than control, irrespective of genotype Null allele LDLR mutations show more severe clinical phenotype in FH patients
Rubba et al. 2017 (18)	Naples, Italy	Retrospective cohort study	LDLR APOB PCSK9	263	Carotid plaques	Plaques were associated with presence of causative mutations. Carotid plaques were significantly associated with premature CVD (OR 4.24, 95%CI 1.29–13.91, *p* = 0.017)
Huijgen et al. 2011 (15)	Netherlands	Cross-sectional study	LDLR APOB	421	Carotid IMT	FH-high (*n* = 162) had higher carotid IMT (0.664 mm) than FH-low (*n* = 114, 0.623 mm, *p* < 0.001) and No-FH (*n* = 145, 0.628 mm, *p* = 0.67)
Vlad et al. 2021 (24)	Romania	Prospective cohort study	LDLR APOB PCSK9	61	Carotid IMT	Pathogenic mutation carriers experienced an increase in cIMT values at 12 and 36 months. Carriers of pathogenic mutations had significantly increased cIMT compared to participants without mutations. APOB and PCSK9 mutations were benign
Bos et al. 2015 (14)	Rotterdam	Cross-sectional study	LDLR APOB PCSK9	191	Carotid IMT	PCSK9 mutations were not found. Patients with high vs. low Lp(a) levels (<0.3 g/L) had similar plaque prevalence (36 and 31%, *p* = 0.4) and C-IMT (0.59 ± 0.12 and 0.59 ± 0.13 mm, *p* = 0.8).
Tada et al. 2023 (25)	Kanazawa University Hospital, Japan	Retrospective cohort study	LDLR APOB PCSK9	622	Coronary and carotid plaque scores	The presence of pathogenic variants was associated with a higher risk of MACEs, with a hazard ratio (HR) of 2.54 and a 95% confidence interval (CI) of 1.34–3.74 (*P* < 0.001).
Noto et al. 2022 (19)	Italy	Retrospective cohort study	LDLR APOB PCSK9	836	Carotid plaques	Carriers of gene variants had OR 3.66 to have plaques compared to non-carriers (95%CI 1.43–10.24)
Semenova et al. 2020 (26)	Moscow, Russia	Retrospective cohort study	LDLR APOB	52	Carotid plaques and carotid atherosclerotic lesions	Pathogenic mutations responsible for the development of monogenic FH were identified in 25 out of 52 probands (48%). Prolonged exposure to LDL-C leads carriers of pathogenic mutations to be more likely to develop severe atherosclerotic lesions and tendon xanthomas.
Cao et al. 2018 (27)	Beijing, China	Cross-sectional study	PCSK9	151	Carotid plaques and coronary, carotid, and femoral atherosclerotic lesions	Patients with coronary and carotid atherosclerotic lesions had significantly higher levels of PCSK9 mutations, whereas those with femoral atherosclerotic lesions showed no difference.

### CIMT in FH patients

The comprehensive review of the studies included into the systematic review revealed larger cIMT in FH patients than controls, however, the magnitude of variation between FH patients and controls were different across studies. For instance, in the study of Huijgen et al. mean cIMT in the FH-high group was 0.664 mm while the patients in the FH-low and no-FH groups had considerably lower mean cIMT values, 0.623 mm and 0.628 mm, *p* < 0.001 and *p* < 0.67 respectively (15). This pattern was constant across most investigations, implying that people with FH have a higher risk of carotid artery atherosclerosis, regardless of their precise genetic abnormalities.

### Impact of genetic mutations on carotid atherosclerosis

Most of the studies focused on LDLR and APOB mutations, or on the combination of all three LDLR, APOB, and PCSK9 mutations. In addition to genetic mutations, inflammatory and lipid biomarkers play an essential role in cardiovascular risk assessment. Several studies have examined their interplay with LDLR mutations. Carriers of LDLR mutations exhibited higher cIMT and increased CRP levels, suggesting a link between genetic predisposition and systemic inflammation (16). The inclusion of biomarkers such as triglyceride/HDL-C ratio and IL-6 may enhance risk stratification beyond genetic factors alone. All studies that investigated LDLR mutations revealed consistent association of these mutations with severe clinical profile in FH patients. Specifically, FH patients carrying these types of mutation demonstrated higher cIMT which increased over time and were directly linked to carotid atherosclerosis progression. However, the severity of those associations varied, with some research showing more significant differences than others. In terms of ApoB and PCK9 mutations, studies revealed mixed findings with some studies showing either no association with cIMT or prevalence of carotid plaque or they had been identified as benign mutations. Recent studies suggest that PCSK9 plays a role beyond lipid metabolism, contributing to endothelial dysfunction by increasing oxidative stress and impairing nitric oxide bioavailability (17). Similarly, ApoB mutations may influence inflammatory pathways, as altered lipoprotein particles can trigger monocyte recruitment and cytokine release (18).

Studies that explored these mutations together as pathogenic mutations, rather than examining them separately, identified a consistent link between presence of these mutations and severe atherosclerotic outcomes. In the study of Noto et al. carriers of these pathogenic mutations had an OR of 3.66 for developing plaques (95% CI 1.43–10.24), as well as a higher risk of severe atherosclerotic lesions and tendon xanthomas (19). A study found that mutation carriers had a higher incidence of major adverse cardiovascular events (MACEs), with a HR of 2.54 (95% CI 1.34–3.74, *p* < 0.001). This highlights the importance of genetic determinants in predicting cardiovascular risk in FH patients.

### Association of plaques with mutations

Several investigations found a substantial association between carotid plaques and causal mutations. One study found pathogenic mutations in 48% of FH probands, and these people had a higher frequency of carotid plaques than those who did not have such mutations. In particular, carriers of pathogenic variants showed an odds ratio of 3.66 for the occurrence of plaques compared to non-carriers (95% CI 1.43–10.24). However, the intensity of this association differed between research, with some demonstrating a stronger link between certain mutations and plaque formation than others.

## Discussion

The current systematic review examined the effect of genetic mutations of LDLR, ApoB, and PCSK9 on the advancement of carotid artery atherosclerosis in patients with FH. The systematic review and analysis of included 9 studies revealed that cMIT values of FH patients were significantly higher than controls, mutations on LDLR gene in FH patients were significantly related with the severe FH phenotype, and the presence of pathogenic mutations resulted in greater incidence of carotid plaques and a higher risk of MACEs. Although ApoB and PCSK9 mutations are less frequent (<5%) among monogenic FH cases, available studies suggest that their impact on atherosclerosis progression may be comparable to LDLR mutations (20). However, due to their low prevalence, fewer studies have comprehensively evaluated their clinical significance in FH patients. Given potential genetic and environmental influences, geographical segregation of FH-related mutations may play a role in phenotype severity, though this was not systematically assessed in the reviewed studies.

The findings from relative studies are largely consistent with the findings of the current study. In the study of Ogura et al. FH patients with higher LDL-C and lower HDL-C levels had larger IMT values. Meanwhile, Bertolini et al. claim that carriers of null variant of mutations had a more severe phenotype compared to defective receptor carriers in terms of not only higher LDL-C levels, but also higher prevalence of tendon xanthomas, coronary heart disease, and carotid arteries atherosclerosis (17, 21).

The complex relationship between LDL receptor mutations and other genes in familial hypercholesterolemia and atherosclerosis could potentially have a wide range of clinical implications, including risk assessment, patient management, and treatment options. Integrating genetic information about FH mutations into risk stratification methods can be useful in terms of predicting cardiovascular outcomes. Patients with LDLR mutations may require more aggressive lipid-lowering therapy, while APOB or PCSK9 mutation carriers could benefit from alternative strategies such as PCSK9 inhibitors or novel RNA-based therapies (19, 28). Identifying high-risk genotypes may inform earlier intervention and tailored monitoring plans. Even if there is a lack of studies examining non-LDLR mutations in FH and their relationship to atherosclerosis, this area may open new opportunities for phenotypic or therapeutic comparison studies. PCSK9 gene, for example, is the latest one to be discovered. Its mutations are relatively rare among FH patients and therefore poorly researched (20). Another important point to note is that for heterozygous FH, despite the higher risk of developing atherosclerosis, it is diverse, and its risk profile is determined not only by cholesterol levels, but also by the presence or absence of other genes and biomarkers (22). Based on the detected mutations of the target genes, healthcare providers can suggest not only medications but also lifestyle changes. People with certain mutations associated with faster progression of atherosclerosis might be advised to follow stricter lifestyle recommendations and start drug treatment early. Such an individualized approach can reduce the negative consequences of genetic predisposition and increase the effectiveness of treatment. By providing patients with knowledge about LDL receptor variants and their relationship to carotid atherosclerosis, more informed decisions about their health can be made. Genetic testing plays a crucial role in diagnosing FH and identifying individuals at risk for early cardiovascular disease. While genetic testing for FH enables early identification of high-risk patients, its routine implementation in clinical practice remains limited due to cost and accessibility constraints. Next-generation sequencing and other molecular techniques are primarily available in specialized centers, and their affordability varies across healthcare systems. Further efforts are needed to integrate these technologies into routine clinical practice.

One limitation of this systematic review is the variability in genetic data reporting among the included studies. While some studies provided general genotype information, the specific mutations and identification techniques were not consistently detailed. While it would be valuable to analyze the specific mutations and their detection methods, the reviewed studies did not consistently report this level of detail. Future research should aim to standardize genetic reporting in FH studies to facilitate cross-study comparisons.

## Conclusion

In conclusion, our results emphasized the important role of mutations, specifically, LDLR mutations in progression of carotid atherosclerosis in FH patients with pathogenic mutations strongly associated with increased plaque formation and cardiovascular risk. These results highlight the significance of tailored treatment plans and genetic screening in the successful management of familial hypercholesterolemia and the reduction of cardiovascular risk.

## Data Availability

The original contributions presented in the study are included in the article/Supplementary Material, further inquiries can be directed to the corresponding author.
